# The role of ideation on the effect of an SBC intervention on consistent bed net use among caregivers of children under 5 years in Nigeria: a multilevel mediation analysis

**DOI:** 10.1186/s12889-021-11709-5

**Published:** 2021-09-13

**Authors:** Osabohien Mathew Okoh, Bolanle Olapeju, Foyeke Oyedokun-Adebagbo, Uwem Inyang, Anna McCartney-Melstad, Ian Tweedie, Stella Babalola, Douglas Storey

**Affiliations:** 1 Johns Hopkins University Center for Communication programs (JHUCCP), Baltimore, USA; 2 United States President’s Malaria Initiative/United States Agency for International Development (PMI/USAID), Washington, D.C., USA

**Keywords:** Mediation, Ideation, Consistent, Net use, SBC, Multilevel, Nigeria

## Abstract

**Background:**

Malaria remains a significant public health challenge in Nigeria. Consistent bed net use (sleeping under a treated net every night) has been identified as a key malaria prevention behavior. This paper explores the relationship between mass media social and behavior change interventions, psychosocial factors, and consistent bed net use.

**Methods:**

Data is from the endline survey of a USAID-funded social and behavior change communication campaign conducted from 2012 to 2017 across five states in Nigeria. The outcome measure was consistent bed net use, and the mediator variable was a composite measure called ideation from a set of psychosocial factors believed to influence bed net use. The independent variable was recall of malaria specific media messages. Multilevel mediation analysis explored if recall of malaria specific media messages had any effect on bed net related ideation and if this ideation had any effect on consistent net use.

**Results:**

Respondents included in this study were on average aged 31 years, mostly married or cohabiting (97.5%) and female 75%. Four in 10 (39.7%) respondents were able to recall malaria specific messages. Respondents with low, moderate and high recall were 23, 32 and 80% more likely to have a higher ideational score in the emotional domain compared to those not able to recall. Respondents were more likely to have higher ideational scores in the cognitive domain if they had low (AOR = 1.26, 95% CI 1.15–1.38), moderate (AOR = 1.16, 95% CI 1.00–1.34) or high recall (AOR = 1.55, 95% CI 1.16–2.06), respectively compared to those with no recall. Similarly, respondents with low (AOR = 1.03, 95% CI .99–1.08), moderate (AOR = 1.15, 95% CI 1.08–1.23) and high (AOR = 1.15, 95% CI 1.01–1.30) recall were more likely to have a higher ideational score in the social domain compared to those with no recall. After adjusting for recall of media messages and other potential covariates, all three ideational domains also had a significant positive effect on consistent bed net use. For every unit increase in ideational score, the likelihood of reporting consistent bed net use increased by 5 to 10%. There was a significant indirect effect of recalling malaria specific messages on consistent bed net use through each of the ideational domains.

**Conclusion:**

Access to a bed net is a critical first step in the process of bed net utilization. However, psychosocial factors e.g., emotional, cognitive, and social domains of ideation also play a major role in bed net use. Mass media SBC interventions could potentially influence bed net related ideation and consequently improve net use behavior. Future Social and behavior change interventions should employ approaches that improve these domains of ideation within their audiences in order to increase bed net utilization.

**Supplementary Information:**

The online version contains supplementary material available at 10.1186/s12889-021-11709-5.

## Background

Malaria remains a significant public health challenge in Nigeria, accounting for 60% of all outpatient hospital visits, 30% of all hospital admissions, 11% of maternal mortality 25% of infant mortality and 30% of under-five mortality [1]. Nigeria has the highest malaria related burden globally. According to the World Health Organization (WHO), Nigeria accounted for 27% of all malaria cases and 23% of malaria related deaths globally in 2017 [2]. This is in spite of the fact that malaria is not only curable but also preventable.

Insecticide Treated Nets and long-lasting insecticidal nets (ITNs) have been shown to be effective in preventing malaria [3, 4]. Yang and colleagues in a recent report also noted that use of ITNs can reduce the incidence of malaria by as much as 41 and 56% respectively [5]. In recognition of the effectiveness of ITNs in preventing malaria, the WHO in 2007 recommended full (universal) coverage of populations at risk of malaria with ITNs [3]. Universal coverage is defined as access to one ITN per every two household members. In order to improve access to ITNs and encourage their use, the government of Nigeria and its development partners distributed 98 million ITNs through free mass distribution campaigns between 2009 and 2015 [1].

Access to an ITN is a necessary first step in the process of using an ITN. Indeed, research has shown that access to an ITN is the most important determinant of net use [6, 7]. Such that the likelihood of sleeping under an ITN increases as the number of accessible ITN is increased in the household [8, 9]. There is evidence, however, that some individuals who have access to ITNs still do not use them [7, 10]. This suggests that there are other factors that play a role in the use of ITNs. Demographic, socioeconomic and psychosocial factors have been identified to influence use of ITNs [9, 11–13]. Besides access to an ITN, ideational factors have also been shown in the literature to influence bednet use [11].

Social and behavior change (SBC) interventions have been used to complement structural and systems-related health interventions to improve and sustain health outcomes at the individual as well as community levels [14]. SBC interventions have also been used to successfully promote adoption and practice of malaria-related behaviors including use of ITNs [11, 15]. Interpersonal communication (IPC), the use of media (e.g., radio, television, print), and community/social mobilization are some examples of SBC approaches that have been used to influence health behaviors [14, 16]. Several authors have reported that SBC interventions act through an intermediary construct called ideation to influence behavior change [12, 17–19].

Ideation is a construct defined as a set of ideas that people hold and that influence how people behave [20, 21]. Ideation as a construct is categorized into three domains and each domain is comprised of a set of psychosocial factors: cognitive (attitudes, knowledge, perceived risk, subjective norms, self-image), emotional (preferences, self-efficacy), and social (social support, social influence, interpersonal communication, personal advocacy) [21–23]. Kincaid (2000) argued that these ideational factors interact cumulatively to influence behavior change because behavioral decisions are rarely influenced by a single factor. The more relevant positive ideational elements an individual has working in their favor, the higher the probability that the individual will adopt the behavior in question [21, 22]. In a five-country study of family planning ideation, Kincaid et al. (2007) found consistent predictive value of ideation on contraceptive behavior across diverse national and programmatic contexts. Other researchers have found similar results for various health behaviors. For example, Storey et al., (2018), Babalola et al., (2015), Ricotta et al. (2015), Russell et al. (2015), Babalola et al. (2016) and Babalola, Vondrasek, Brown, & Traoré; (2001) have all independently demonstrated the power of a person’s ideational profile to predict behavior [12, 13, 18, 23, 25, 26].

Whereas there is evidence of a direct effect of SBC intervention on health behaviors [11, 27, 28], many researchers have argued that SBC interventions bring about behavior change by first changing ideation which in turn influences adoption of health behaviors [12, 17–19], necessitating the need to deconstruct the relationship between SBC intervention and ideation in the process of behavior change. According to VanderWeele (2015) the process by which an exposure leads to an outcome via an intermediate factor is called mediation and the technique of separating the effect of the exposure and the intermediate factor on the outcome is called mediation analysis [29].

Mediation analysis has been used extensively to demonstrate the direct and indirect effect of exposure on outcomes in different areas, including education and health [30], quality of antenatal care [31], financial planning [32], nutrition [33], disease causation [34–36], and disease management [37]. However, there has been limited use of this technique in the field of SBC [19, 38], particularly with respect to malaria prevention and treatment behaviors [18]. This study is therefore an attempt to contribute to this body of knowledge by describing the relationship between SBC intervention, ideation and consistent use of ITNs (sleeping under a net every night) among residents of three Nigerian States (Akwa Ibom, Kebbi and Nasarawa), where the Health Communication Capacity Collaborative (HC3) project was implemented.

The USAID funded HC3 project was a five-year (2012–2017) project implemented in five states that aimed to improve the practice of key malaria prevention and treatment behaviors (such as the consistent use of ITNs, uptake of intermittent preventive treatment of malaria in pregnancy (IPTp), and prompt and appropriate management of fever across the five intervention states). The project designed and implemented a set of SBC interventions (aimed at improving the ideational factors believed to influence malaria behavior adoption/practice) that included mass media messaging, interpersonal communication and community mobilization. This study assessed the effects of the mass media component of the interventions, which included radio and TV spots in English, Pidgin, Hausa, and other local languages in each state; weekly radio magazine shows (“*Play Your Part”* in Pidgin and English*; Taka Naka Rawan* in Hausa, and locally produced shows in local languages in selected focal states); a national malaria theme song *(“Play Your Part”)* and the *Newman Street* television drama series. In all, there were 15 different media materials that respondents could have been exposed to. Specifically, we examined the pathway through which the HC3 SBC intervention influenced consistent use of ITNs (sleep under bed net every night).

### The HC3 SBC intervention

#### Radio and TV spots/jingles

The HC3 project produced and aired a series of one-minute radio spots (in English and local languages) on television and radio. These jingles promoted appropriate malaria-related behaviors including, use of ITNs, prompt care-seeking for fever in a health facility, use of diagnostic tests before treating malaria, and prevention of malaria in pregnancy. These jingles/spots were broadcast multiple times a day on two radio and two TV stations in each State and were on air for about 18 months before the survey.

#### Radio magazine shows

These were 20-min radio shows titled “*Play Your Part”* in English and Pidgin languages and *“Taka Naka Rawan”* in the Hausa language that were aired on a weekly basis. These programmes were further adapted to fit the state context in the five project States including Akwa Ibom, Kebbi, and Nasarawa. The radio shows essentially disseminated the same messages as the radio and TV spots.

#### Malaria theme song

*“Play Your Part”* featuring top Nigerian musicians was developed and provided the theme music for the spots and radio shows, as well as a standalone song and music video. Again, this song promoted the adoption of appropriate malaria behaviors, including the use of an ITN every night.

### Newman street TV Drama

In October 2014, HC3 and its partners launched a television drama series called *Newman Street*. The television drama was designed to influence adoption of recommended malaria and other health behaviors. In addition to the weekly airing of *Newman Street* on different TV stations, HC3 also screened malaria specific excerpts of *Newman Street* during community events to improve exposure to its contents.

The underlying conceptual framework for this research is the ideation framework, a meta-theoretical model of behavior change, which has been used to understand, predict, and influence a range of health behaviors [11, 21, 22]. The Ideation model includes concepts borrowed from multiple behavioral theories, including the Health Belief Model, the Theory of Planned Behavior, The Extended Parallel Process Model and the Social Cognitive Theory. The model includes three domains of psychosocial factors, cognitive, emotional and social interactions. The cognitive domain refers to elements that are involved in understanding of the behavior and its perceived positive or negative consequences. These elements include knowledge, beliefs, values, perceived risk, subjective norms and self-image. The emotional domain refers to elements that relate to the feelings associated with the behavior. Such elements include perceived self-efficacy, emotional response, and empathy. The social domain comprises of the interpersonal relationships and influences from family, friends, and community. This domain includes elements such as personal advocacy, discussion of the behavior with others, perceived social support, and descriptive norm.

We posit that all three domains of ideation individually and synergistically act as intermediate outcome variables that influence bed net utilization.

## Objective

Our main objective for this study was to identify the relationship between HC3’s mass media SBC interventions, ideation, and consistent bed net use.

### Hypothesis

We hypothesized that recall of the HC3 mass media SBC messaging will indirectly influence the consistent use of a bed net (ITN) through its effect on net use-related ideation.

The concept of indirect effect is not new in the literature. Research has shown that exposure variables can indirectly influence behavioral outcomes through their effects on some other mediating variables [39–41].

## Methods

### Study setting

Data were collected from three Nigerian States of Akwa Ibom, Kebbi, and Nasarawa. The three states are located in the South-South, North-West and North-Central geopolitical zones in Nigeria respectively. The dominant ethnic groups in Akwa Ibom are Ibibio, Annang, and Oron. While Hausa, Fulani, Dakarki and Kamberi are the dominant ethnic groups in Kebbi. Eggon, Afa, Alago, Mada, and Koro are the dominant groups in Nasarawa State. Akwa Ibom State is predominantly populated by Christians, While Kebbi State is predominantly populated by Muslim State. Nasarawa State on the other hand, has a mix population of Christian and Muslim. As in any Nigerian State, malaria is endemic to these three states. Educationally, 5.4% of women aged 15–49 years have no education in Akwa Ibom compared to 29.5% in Nasarawa and 71.9% in Kebbi. According to the most recent malaria indicator survey in Nigeria, Kebbi State has the highest proportion of households with at least one ITN at 86.7%, while this proportion is 76.4% in Nasarawa and 74.2% in Akwa Ibom State. Similarly, prevalence of parasitemia in children was highest in Kebbi at 63.6, 35.9% in Nasarawa and 22.8% in Akwa Ibom [1].

### Sampling

The USAID funded HC3 Nigeria project was evaluated using a quasi-experimental design with a pre-intervention (baseline) and a post-intervention (endline) survey conducted in Akwa Ibom, Kebbi and Nasarawa states. The present study is based on data derived from the endline survey. Study participants were selected through a multi-stage process that involved successively and randomly selecting LGAs, clusters, households and individuals after a complete mapping and listing of all households in the selected clusters.

Firstly, six local government areas (LGAs) were selected from each state using probability proportional to size sampling technique. Secondly, ten clusters (census enumeration areas) were then selected from among the existing list of census enumeration areas in each selected LGA using a systematic sampling approach for a total of 60 clusters in each state. Thirdly, complete mapping and listing of all households in the selected clusters was conducted. During the mapping and listing exercise, households with at least one child under the age of 5 years and a female caretaker aged 15–49 years were identified. Only households that met these criteria were included in the final sampling frame from which 20 households were randomly selected for survey participation. Finally, a child 0 to 4 years resident in the households sampled were randomly selected and their mother or female caretaker aged 15–49 years were requested to participate in the interview.

We determined the required sample size for this study based on the proportion of the population with positive attitudes towards bed nets. Since we did not have such information for any of the study states, we assume that this indicator is 50%. This level of prevalence of positive attitudes is ideal for estimating the required sample size as it provides maximum variability [42, 43]. In addition, we set an effect size target namely that program activities would result in an increase of 10 percentage points in the prevalence of positive attitudes towards bed nets. This is in line with effect sizes that have been established in earlier studies [44, 45]. We applied the following formula to determine the sample size:

$$ n=\frac{{\left({Z}_{\alpha}\sqrt{2p\left(1-p\right)}+{Z}_{1-\beta}\sqrt{p_1\left(1-{p}_1\right){p}_2\left(1-{p}_2\right)}\right)}^2}{{\left({p}_1-{p}_2\right)}^2} $$

Where: *p*_1_ and *p*_2_ are the proportions with the desired outcome at baseline and endline, respectively; *and, Z*_a_ and *Z*_1-b_ are normal deviates.

Assuming a power of 0.90 and design effect of 2.0, we estimated the required number of households to sample to be 1200 in each state.

### Data collection

We implemented data collection in September 2017 among a sample of men and women of reproductive age (18–49 for women and 18–59 for men). The respondents were selected from households with at least one child aged less than 5 years old.

In the households selected for the survey and in which the head of household had consented to participate in the survey, the fieldworker first administered the household questionnaire. The household questionnaire included a listing of all members of the household. Using that listing, the fieldworker randomly selected a child between the ages of 0 and 4 years from the list and identified the child’s mother for inclusion in the survey. Following the completion of the household questionnaire, the fieldwork team approached the female caretaker selected for the individual questionnaire and administered the questionnaire if she gave consent to participate. In every third household that agreed to participate in the survey, the fieldwork team also identified the spouse of the selected female caretaker and interviewed him with the individual questionnaire if he gave consent.

Interviews were completed in 3555 households from which 3528 female caregivers and 1155 spouses completed the survey across the three study states. However, only 2745 respondents from households with at least one bed net were included in the analysis for the current study. Since people living in households without bed nets cannot be reasonably expected to sleep under a bed net, they were all excluded from the analysis. Hand-held devices were used to collect and transmit data to an online folder designed for the study.

### Data processing and analysis

#### Outcome (dependent) variable

The main outcome measure was consistent bed net use, a binary variable defined as sleeping under a bed net every night versus not sleeping under a bed net every night. Respondents were asked how often they slept under an insecticide-treated bed net. The question was worded as follows: In general, how often do you sleep under a mosquito net – every night, most nights, some nights, very few nights or never?. Those who self-reported that they slept inside a bed net every night were coded as consistent net users. While respondents who reported otherwise were coded as not using a bed net consistently.

#### Mediator variable

We created a composite variable called ideation from a set of psychosocial factors believed to influence bed net use. The psychosocial factors examined in this study were:

Cognitive elements

1. Knowledge about the cause of malaria
2. Knowledge of a place to purchase bed nets
3. Knowledge that use of bed net is a way to prevent malaria
4. Attitudes towards bed nets
5. Descriptive norm about bed net use
6. Perceived severity of malaria, if infected
7. Perceived susceptibility to malaria infection
8. Perceived response-efficacy of bed nets (belief that sleeping inside a bed net can prevent malaria infection).

Emotional elements

9. Perceived self-efficacy to prevent oneself or one’s children from malaria
10. Perceived self-efficacy to procure and use bed nets
11. Willingness to pay for nets

Social elements

12. Discussion about bed nets with others
13. Participation in net allocation decisions in the household

Details of how these factors were measured and manipulated are described in appendix 1.

#### Explanatory (independent) variable

The main explanatory variable examined in this study was recall of the malaria specific media messages. This variable has four categories that include no recall (people who were not able to recall any of the messages), low recall (respondents who were able to recall between one and five messages), moderate recall (respondents who were able to recall between six and 10 messages) and high recall (respondents who were able to recall between 11 and 15 messages). The respondents were asked specifically about exposure to any of the 15 different media messages that the USAID funded HC3 project produced and aired. For example, respondents were asked “In the last six months, did you hear a jingle on the radio or the television with Oga Bulus talking about how people can be free of malaria?” or In the last 6 months, did you hear a jingle on the radio or the television with Mallam Bala talking about how people can be free of malaria? Oga Bulus was the main character in the jingles produced in English or Pidgin English languages while Mallam Bala was the main character in the Hausa language versions of the jingles. Respondents who answered “No” will then be asked to listen to shortened version of the jingle (reduced to 20 s) such that it helps to jug the memory of the respondents without revealing the message of the jingle. After listening to this short version, the respondent will then be asked if he or she has heard that jingle before. Those who respond in the negative are then asked about exposure to another jingle. Those who answered in the positive, either to the unaided question or after listening to the shortened version are then asked to state the main message of the jingle. Only respondents who were able to recall the message of each specific jingle were categorized as being able to recall the messages for such jingles. We did not ask if respondents were exposed to specific messages multiple times. This is also noted in the limitation section.

#### Other independent variables (covariates)

The fitted models adjusted for the following covariates:

- Sociodemographic characteristics: gender (defined as male or female), age (continuous), level of education (categorized as no education, primary education, secondary education and tertiary education), and religion (categorized as Christian and others).
- Household variables: household wealth quintile (an asset-based construct), access to a bed net within the household (binary variable, defined as living in a household where there was at least one bed net for every two people).
- Community variables: type of place of residence (defined as rural or urban, state of residence (categorical variable representing each of the three states included in the study, and intervention status of the community (binary variable, defined as living in a cluster where interpersonal and /or community mobilization took place).

#### Analysis

Given that the intervention was aimed at influencing psychosocial factors which were in turn expected to affect net use, we therefore conducted a mediation by design analysis [46, 47] to examine if recall of USAID funded HC3 malaria specific media messages had any effect on bed net related ideation and if ideation had any effect on consistent net use after adjusting for other intervention component. The outcome variable, consistent net use, and all 13 psychosocial factors (see appendix 1 for details) included in the analysis were dichotomous. First, a multivariate logistic regression model (adjusted for covariates described above) was fitted to determine which of the psychosocial variables were positively associated with the outcome variable at *p* < 0.05 level. We then created a composite variable called ideation by summing the score of all nine psychosocial factors (knowledge of a place to purchase bed nets, knows that use of bed net is a way to prevent malaria, descriptive norm about bed net use, discussion about bed nets, willingness to pay for nets, perceived susceptibility to malaria, attitude towards bed net, perceived self-efficacy to procure and use bed nets as well as perceived self-efficacy to protect self and children from malaria) that were associated with the outcome variable from the fitted logistic model for each respondent. The value of the composite variable ranged from 1 to 9, and the higher the value of the index, the higher the respondent’s ideational profile.. Similarly, we also created three index variables that correspond to the cognitive, emotional, and social domains of the ideation model by summing the scores of the relevant psychosocial factor for each domain that were positively associated with the outcome variable (psychosocial factors for each ideation domain are described above as well as in appendix 1). The cognitive domain being composed of five psychosocial factors had a score of between 0 and 5, while the emotional domain had a range of 0–3 since three factors were used to create this domain. The social domain was created with only one factor since the second factor was not significantly associated with the outcome variable. In order to account for the clustered nature of our data (respondents grouped into 179 clusters), we conducted multivariate multilevel mediation analyses to estimate the net effect of the intervention on the outcome variable and at the same time identified the pathway through which the intervention influenced the outcome while simultaneously accounting for the unmeasured community level factors that could have influenced the outcome variable. Multilevel modeling is ideal for this analysis because it helps to systematically account for the influence of factors at different levels on the outcome as well as accounting for the similarities among respondents from the same community (cluster). Generalized structural equation modeling (*gsem*) command was used for our model estimation because it is robust enough to allow for multilevel analysis as well as supports a binary outcome variable. Indirect effects were estimated using the non-linear combination (*nlcom*) command because we estimated a combination of linear and non-linear transformations.

We conducted two multilevel mediation analyses, the first model included only the composite variable “ideation” as the mediator, while the second model was a multiple mediator model that included the three index (cognitive, emotional and social domains of ideation) variables without the composite variable. All analyses and data processing were performed using Stata/SE 16.0 for windows. Results were interpreted at *p* < 0.05.

## Results

While the original data included 4683 (3528 female caretakers of children under 5 years of age and 1155 spouses) respondents. This current study included 2745 (2059 female caretakers of children under 5 years of age and 686 spouses) respondents from households with at least one bed net. Respondents included in this study were on average aged 31 years (18–59 years), mostly married or cohabiting (97.5%) and female 75%. The majority lived in a rural setting (80.7%), had less than a secondary education (58%), and practiced the Christian faith (52.6%). Four in 10 (39.7%) respondents recalled at least one of the HC3 project’s malaria specific messages. Of those who recalled the messages, most (72%) of the respondents had low recall (recalled one to five messages), less than one-quarter had moderate recall (six to 10 messages) while 5% had high recall (11 to 15 messages) of the media messages. Details of respondents’ background characteristics are presented in Table 1.

**Table 1:** Percent distribution of respondents from households with at least one net, by socio-demographic, psychosocial and household characteristics; Nigeria 2017

Background characteristics	State			
	Akwa Ibom (***n*** = 845)	Kebbi (***n*** = 964)	Nasarawa (***n*** = 936)	Total (***n*** = 2745)
**Age group**				
18–24	18.34	21.78	19.55	19.96
25–34	48.17	46.58	52.88	49.22
35–44	26.15	22.10	21.05	22.99
45 and above	7.34	9.54	6.52	7.83
**Respondent’s sex**				
Male	25.68	24.17	25.21	24.99
Female	74.32	75.83	74.79	75.01
**Marital status**				
Never married	2.37	0.10	0.64	0.98
Married/co-habiting	94.79	99.27	97.97	97.45
Divorced/separated/widowed	2.84	0.62	1.39	1.57
**Educational level**				
None	3.79	77.18	36.11	40.58
Primary	24.02	8.20	21.15	17.49
Secondary	58.58	9.96	35.36	33.59
Higher	13.61	4.67	7.37	8.34
**Religion**				
Christian	99.76	7.47	56.52	52.60
Muslim	0.24	89.94	41.56	45.83
Traditional	0	2.59	1.92	1.57
**Place of residence**				
Rural	81.54	77.07	83.76	80.73
Urban	18.46	22.93	16.24	19.27
**Wealth index**				
Lowest	2.96	32.26	11.43	16.14
Second	8.28	24.27	23.40	19.05
Middle	18.82	17.12	26.71	20.91
Fourth	31.72	11.10	19.23	20.22
Highest	38.22	15.25	19.23	23.68
Average household size	5	6	6	6
Average number of nets owned per household	2	2	2	2
Proportion residing in households with at least one net for two residents	32.02	33.51	17.84	28.01
**Psychosocial profile**				
knows that malaria is caused by mosquito	31.24	70.54	59.40	54.64
Higher perceived self-efficacy to protect self and children from malaria	52.66	45.75	49.15	49.03
Higher perceived susceptibility to malaria	52.43	42.74	48.72	47.76
Higher perceived severity of malaria	37.51	50.00	46.15	44.85
Knows that bed net prevent malaria	72.66	89.63	86.54	83.35
Higher perceived self-efficacy to procure and use a bed net	25.68	43.78	36.11	35.59
Had discussion about bed net	21.18	51.66	46.79	40.62
Participates in decision about net allocation	64.73	57.57	62.82	61.57
Higher perceived response efficacy of bed net	36.69	41.18	49.04	42.48
Perceived that use of bed net is a norm	33.61	72.82	61.86	57.01
Knows where to buy a bed net	14.79	43.78	33.55	31.37
Willing to pay for a bed net	31.38	63.07	71.15	56.21
Positive attitude towards bed net	34.67	42.32	42.74	40.11

More than four-fifth (83.35%) of our respondents knew that bed net can prevent people from getting malaria. Close to two-thirds (61.57%) reported that they participate in decisions on how bed nets are allocated in their household. While a little more than half (57%) thought that at least half the people in their community use a bed net (see psychosocial profile section of Table 1). Of the psychosocial factors measured by Likert scale questions, perceived response efficacy of bed net, perceived self-efficacy to procure and use bed net, as well as perceived self-efficacy to protect self and children from malaria had the highest mean score of 6, 4 and 2.6 respectively.

### Bed net ownership and use

The average number of bed nets owned was two nets per household. Less than three in 10 (28%) respondents resided in households that owned one net for every two persons (universal bed net coverage). Overall, consistent bed net use was reported by half (51%) of the respondents. This proportion increased to 59% among respondents residing in households with universal bed net coverage. Details of consistent bed net use by respondent’s demographic and psychosocial characteristics are shown in Table 2.

**Table 2:** Bivariate analysis of consistent net use by respondents’ characteristics (*n* = 2745)

Background characteristics	Consistent bed net use		***P*** value
	Yes	No	
**Age group**			
18–24	57.48	42.52	
25–34	52.04	47.96	
35–44	45.48	54.52	
45 and above	44.19	55.81	*P* < 0.001
**Respondent’s sex**			
Male	40.38	59.62	
Female	54.54	45.46	*P* < 0.001
**Marital status**			
Never married	51.85	48.15	
Married/co-habiting	51.21	48.79	
Divorced/separated/widowed	37.21	62.79	*P* = 0.189
**Educational level**			
None	56.19	43.81	
Primary	51.25	48.75	
Secondary	46.53	53.47	
Higher	43.23	56.77	*P* < 0.001
**Religion**			
Christian	43.56	56.44	
Muslim	59.78	40.22	
Traditional	44.19	55.81	*P* < 0.001
**Place of residence**			
Rural	48.96	51.04	
Urban	51.49	48.51	*P* = 0.296
**Wealth index**			
Lowest	50.79	49.21	
Second	56.41	43.59	
Middle	52.79	47.21	
Fourth	49.55	50.45	
Highest	46.46	53.54	*P* = 0.013
**Psychosocial profile**			
**Knows that malaria is caused by mosquito**			
Yes	57.33	42.67	
No	43.37	56.63	*P* < 0.001
**Higher perceived self-efficacy to protect self and children from malaria**			
Yes	57.80	42.20	
No	44.46	55.54	*P* < 0.001
**Higher perceived susceptibility to malaria**			
Yes	44.70	55.30	
No	56.76	43.24	*P* < 0.001
**Higher perceived severity of malaria**			
Yes	52.72	47.28	
No	49.60	50.40	*P* = 0.104
**Knows that bed net prevent malaria**			
Yes	54.06	45.94	
No	35.67	64.33	*P* < 0.001
**Higher perceived self-efficacy to procure and use a bed net**			
Yes	65.10	34.90	
No	43.21	56.79	*P* < 0.001
**Had discussion about bed net**			
Yes	60.45	39.55	
No	44.54	55.46	*P* < 0.001
**Participates in decision about net allocation**			
Yes	50.83	49.17	
No	51.28	48.72	*P* = 0.818
**Higher perceived response efficacy of bed net**			
Yes	56.35	43.65	
No	47.06	52.94	*P* < 0.001
**Perceived that use of bed net is a norm**			
Yes	59.36	40.64	
No	39.92	60.08	*P* < 0.001
**Knows where to buy a bed net**			
Yes	63.65	36.35	
No	45.22	54.78	*P* < 0.001
**Willing to pay for a bed net**			
Yes	58.26	41.74	
No	41.68	58.32	*P* < 0.001
**Positive attitude towards bed net**			
Yes	59.58	40.42	
No	45.26	54.74	*P* < 0.001

### Mediation analysis

**Fig. 1:**
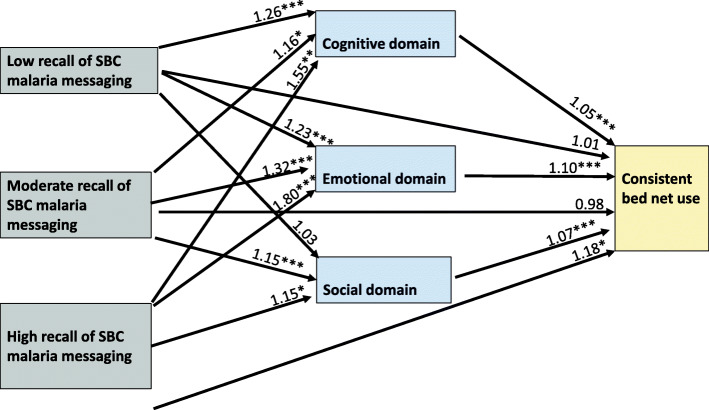
Multiple mediation pathway for the effect of HC3 malaria SBC media exposure on consistent bed net use. *P < 0.05 **P < 0.01 ***P < 0.001

In the model that examined the relationship between our explanatory variable, our hypothesized mediators (domains of ideation) and consistent bed net use, respondents who recalled HC3 media messages were more likely to have higher cognitive, emotional, and social ideation domain scores compared to those not able to recall any message (See Fig. 1 and Table 3). Respondents with high recall were 80% more likely to have a higher ideational score in the emotional domain compared to those not able to recall (AOR = 1.80, 95% CI 1.37–2.34; *P* < 0.001). Respondents with low and moderate recall were 23% (AOR = 1.23, 95% CI 1.13–1.34; *P* < 0.001) and 32% (AOR = 1.32, 95% CI 1.15–1.51; *P* < 0.001) more likely to have a higher score in the emotional domain compared to those not able to recall the message. Respondents were 26, 16 and 55% more likely to have higher ideational scores in the cognitive domain if they had low (AOR = 1.26, 95% CI 1.15–1.38; *P* < 0.001), moderate (AOR = 1.16, 95% CI 1.00–1.34; *P* = 0.043) and high recall (AOR = 1.55, 95% CI 1.16–2.06; *P* = 0.003) respectively compared to their counterparts who did not recall. Similarly, respondents with, moderate, and high recall were 15% (AOR = 1.15, 95% CI 1.08–1.23; *P* < 0.001), and 15% (AOR = 1.15, 95% CI 1.01–1.30; *P* = 0.038) more likely to have a higher score in the social domain compared to those not able to recall. Respondents with low recall, were not significantly differently from those not able to recall any message in this domain. Having adjusted for recall of media messages and other potential covariates, all three ideational domains also had a significant positive effect on consistent bed net use. For every unit increase in ideational score in the emotional, cognitive and social domains the odds of reporting consistent bed net use increased by 10% (AOR = 1.10, 95% CI 1.08–1.12; *P* < 0.001), 5% (AOR = 1.05, 95% CI 1.03–1.07; *P* < 0.001) and 7% (AOR = 1.07, 95% CI 1.03–1.11; *P* < 0.001) respectively. There was a significant indirect effect of the explanatory variable on consistent bed net use through each of the ideational domains. A total of 51.1% of the indirect effect was transmitted through these mediators, with the emotional domain accounting for 31.9%, the cognitive domain accounting for 12.5% and the social domain accounting for 6.7%. After accounting for these mediators, we still found a statistically significant direct effect of the explanatory variable on the outcome variable. However, only respondents with high recall had a 18% higher likelihood of consistent bed net use (AOR = 1.18, 95% CI 1.04–1.34; *P* = 0.011) compared to those not able to recall.

**Table 3:** Multilevel mediation analysis of factors independently associated with consistent bed net use

Respondents’ characteristics	Bivariate regression with unadjusted odds ratio	Model 1 with ideation as a single mediator variable	Model 2 with domains of ideation as separate mediator variables
	COR (95%(CI)	AOR (95% CI)	AOR (95% CI)
Ideation as a composite index	1.44 (1.37–1.50) ***	1.07 (1.06–1.08) ***	
**Domains of ideation**			
Emotional domain	1.64 (1.51–1.77) ***		1.10 (1.08–1.12) ***
Cognitive domain	1.46 (1.36–156) ***		1.05 (1.03–1.07) ***
Social domain	1.90 (1.63–2.22) ***		1.07 (1.03–1.11) ***
**Level of message recall**			
No recall (ref)	1.00	1.00	1.00
Low recall	.97 (.82–1.15)	1.01 (.97–1.06)	1.01 (.97–1.06)
Moderate recall	.82 (.63–1.08)	.99 (.92–1.05)	.98 (.92–1.05)
High recall	2.65 (1.43–4.93) **	1.19 (1.04–1.35) **	1.18 (1.04–1.34) *
**Respondent resides in a location that received community intervention**			
No (ref)		1.00	1.00
Yes		1.05 (0.98–1.12)	1.05 (0.98–1.13)
**Wealth index**			
Poorest (ref)		1.00	1.00
Second		1.06 (1.00–1.13)	1.06 (1.00–1.13)
Middle		1.04 (.97–1.11)	1.04 (.97–1.11)
Fourth		1.05 (.98–1.23)	1.05 (.97–1.12)
Richest		1.03 (.95–1.11) *	1.03 (.95–1.11)
**State of residence**			
Akwa Ibom (ref)		1.00	1.00
Kebbi		1.11 (1.01–1.23) *	1.12 (1.01–1.24) *
Nasarawa		1.10 (1.01–1.19) *	1.10 (1.01–1.19) *
Age		1.00 (0.99–1.00) *	1.00 (0.99–1.00) *
**Type of place of residence**			
Rural (ref)		1.00	1.00
Urban		.94 (.86–1.02)	.94 (.86–1.02)
**Education level**			
None (ref)		1.00	1.00
Primary		1.05 (.99–1.11)	1.05 (.99–1.11)
Secondary		1.04 (.98–1.10)	1.04 (.98–1.10)
Tertiary		1.01 (.93–1.10)	1.01 (.93–1.10)
**Religion**			
Other religion (ref)		1.00	1.00
Christian		.97 (.91–1.04)	.97 (.91–1.04)
**Respondent resides in household with one net for every two residents**			
No (ref)		1.00	1.00
Yes		1.11 (1.06–1.15) ***	1.11 (1.06–1.15) ***
**Respondent’s sex**			
Male (ref)		1.00	1.00
Female		1.15 (1.10–1.20) ***	1.15 (1.10–1.21) ***
**Effect of level of recall on ideation**			
No recall (ref)		1.00	
Low recall		1.54 (1.34–1.78) ***	
Moderate recall		1.71 (1.37–2.14) ***	
High recall		3.03 (1.95–4.70) ***	
**Effect of level of recall on emotional domain of ideation**			
No recall (ref)			1.00
Low recall			1.23 (1.13–1.34) ***
Moderate recall			1.32 (1.15–1.51) ***
High recall			1.80 (1.37–2.34) ***
**Effect of level of recall on cognitive domain of ideation**			
No recall (ref)			1.00
Low recall			1.26 (1.15–1.38) ***
Moderate recall			1.16 (1.00–1.34) *
High recall			1.55 (1.16–2.06) **
**Effect of level of recall on social domain of ideation**			
No recall (ref)			1.00
Low recall			1.03 (.99–1.08)
Moderate recall			1.15 (1.08–1.23) ***
High recall			1.15 (1.01–1.30) *

**Fig. 2:**
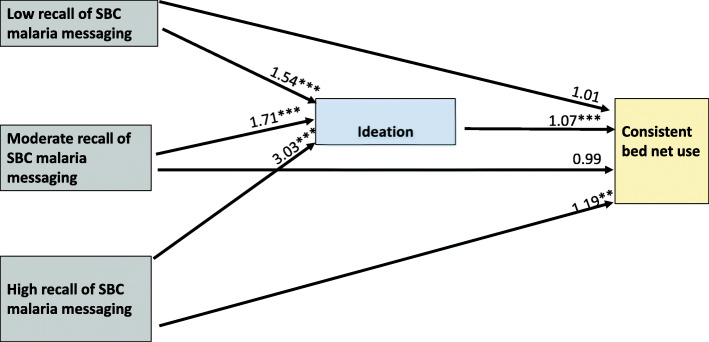
Mediation pathway for the effect of HC3 malaria SBC media exposure on consistent bed net use. *P < 0.05 **P < 0.01 ***P < 0.001

***p < 0.001, **p < 0.01, *p < 0.05,

In the model that included ideation as a single variable of all the three domains, recall of HC3’s malaria media message was also associated with a statistically significant higher ideational score across all three recall levels (see Fig. 2 and Table 3). Respondents were 54% (AOR = 1.54, 95% CI 1.34–1.78; *P* < 0.001), 71% (AOR = 1.71, 95% CI 1.37–2.14; *P* < 0.001), and three times (AOR = 3.03, 95% CI 1.95–4.70; *P* < 0.001), more likely to have a higher ideational score if they had low, moderate or high recall respectively compared to those not able to recall. Furthermore, after adjusting for recall of media messages, a unit increase in ideation score was associated with an increased likelihood of consistent net use (AOR = 1.07, 95% CI 1.06–1.08; *P* < 0.001). In this model, we also found a significant direct effect of our explanatory variable on the outcome. Again, this was only among respondents with high recall (AOR = 1.19, 95% CI 1.04–1.35; *P* = 0.009).

## Discussion

Mediation analysis has been used in other health areas to demonstrate how interventions lead to outcomes through an intermediate (mediator) variable [18, 19, 38]. However, this is the first study to use mediation analysis to investigate the link between SBC messaging and consistent bed net use through a mediating variable called ideation. While access to a bed net is a critical first step in the process of bed net utilization, it is also important to consider other factors like an individual’s psychosocial profile (attitudes, beliefs and social considerations with respect to malaria and bed nets), also referred to as ideation, that play a role in bed net use.

In this study, we used mediation analysis and ideational theory to investigate how SBC interventions using mass media influenced consistent bed net use among caretakers of children under the age of five. This group of individuals are often responsible for multiple children in their care, understanding the ideational factors that influence net use decisions for these people and the children under their care can have broad impact on the health of the entire household.

Our results indicated that the interventions significantly influenced our hypothesized mediator “ideation” both as a composite variable and when deconstructed into its three domains. Respondents who recalled SBC messages were up to three times as likely to have higher ideational scores compared to their counterparts who were unable to recall the messages. This suggests that our indirect effects hypothesis (that the intervention will affect our hypothesized mediator, which in turn will affect the behavioral outcome) was supported. The fact that the odds of higher ideation was consistently highest among those with high recall is worthy of note. If a programmatic goal is to reach more people with effective messaging, then the intervention could be revised to include additional or stronger messaging components that strengthen ideational variables shown to be associated with bed net use [18] and deliver them to more people through the media with the greatest potential to disseminate new ideas, i.e., mass media [22, 24]. Furthermore, the observed dose-response effect suggests that exposing respondents to a higher number of messages could also lead to stronger ideational changes. Such strategies would be likely to produce larger changes in the mediator variable and larger subsequent changes in the behavioral outcome. We were not able to assess the role that frequency of exposure to specific messages had because we did not collect data that would have helped us make such a determination. Furthermore, we were not able to compare our findings with those of other studies, since this is the first study to evaluate the role of a mediator in net use behavior.

This study demonstrated the overall role of ideation as well as its comprising elements in improving bed net uptake. Communication influences each of these elements separately, as well as jointly as these ideational elements occur simultaneously and are interdependent. One programmatic implication of this study finding is that social and behavior change interventions should employ messaging that not only improves knowledge about bed net use and its consequences, but promotes a positive attitude about bed nets, generates conversation regarding the use of bed nets and also enables the audience to feel right about it.

As ideation increased, the likelihood of consistent bed net use also increased. When the different ideational domain scores were considered, the emotional component appeared to have the largest effect of the three followed by the social domain. This means that people with higher ideational scores in the emotional domain had the highest odds of consistent bed net use. All three ideational domains, had a dose-response relationship with levels of recall of messages. The higher the level of recall, the higher the ideational score in each domain. However, emotional and social ideational domains accounted for almost all the indirect effect transmitted to the outcome variable. Mass media can have extremely powerful social and emotional effects if the messages are designed to do so (e.g., by encouraging and modeling discussion with peers or by creating empathy for people trying to protect their family from malaria). Thirty- to sixty-second TV and radio spots may be limited in terms of how much content they can deliver in that short span of time, but longer formats like serials, documentaries, or radio diaries are far less limited in that regard and can depict people struggling with a decision, trying, failing, and then overcoming barriers to succeed [48, 49].

When ideation was considered as a composite variable, it also exhibited a significant positive effect on bed net use. Thereby confirming our conceptual theory that a change in the hypothesized mediator will lead to a change in the outcome, and consequently validating our theoretical assumptions. After adjusting for potential covariates including ideation and clustering, there was a significant positive direct effect of recall of SBC messaging on consistent bed net use. This suggests that our hypothesized mediator did not completely account for the effect of our explanatory variable on the outcome. Thereby implying the likely presence of other mediating factors that we did not account for. Baron and Kenny (1986) described this scenario as a partial mediation while Zhao, Lynch JR & Chen, 2010 described it as complementary mediation [39, 40]. Nonetheless, our hypothesized mediator transmitted 51.1% of the indirect effect of our intervention to the outcome variable.

### Limitations

Although we implemented a quasi-experimental intervention, the data analyzed for this study was cross-sectional and so it limits the extent to which causality can be claimed. However, the observations are conceptually plausible, and identify a conceptual framework for subsequent evaluation of SBC interventions, particularly in the field of malaria programming. Since the data used for this study were self-reported, we are not sure how social desirability bias may have influenced our findings. Nonetheless, the dose-response effect observed with increasing levels of recall lays credence to a possible causal mechanism. Furthermore, the rigorous and robust analytical techniques employed in this study provide a strong support for the results presented.

### Implications for SBC programming

Findings from this study have a few implications for SBC programming. The fact that ideation increased in direct relation to message recall, suggests that SBC programmers might consider use of multiple messages in interventions aimed at improving the use of bed nets. More importantly, communication interventions aimed at improving bed net use should consider messages that appeal to the emotional domain (e.g., self-efficacy) such as “It is easy to procure and use bed nets because they are widely available from pharmacies and often are distributed free at health facilities.” . Furthermore, messages should be designed to promote the fact that bed nets effectively prevent malaria and that most people in the community use a bed net.

## Conclusions

Findings from this study suggests that the use of mass media could be effectively used to influence the attitude, perceptions, beliefs, values, emotional and social considerations (ideation) that people hold concerning ITNs, thereby increasing the likelihood of consistent bed net use. In addition, our study further lays credence to the utility of the ideation theory in the evaluation of SBC interventions and how ideation is a pathway through which such interventions influence behaviors. Mediation analysis is a useful technique for deconstructing the relationship between SBC interventions, mediator variables and behavior, thereby providing insight into which mediators should be targeted for a more effective design and implementation of SBC interventions. Expanded use of this technique is therefore warranted in malaria SBC programming, especially for the population included in this analysis. The emotional domain of ideation as the greatest influencer of consistent bed net use in the population of interest should be prioritized in future programming. The use of media channels that reach greater numbers of people with greater frequency might help to enhance message impact on emotional aspects of ideation and its subsequent impact on behavior. Programmers should also not ignore the cognitive domain as a significant portion of the indirect effect was transmitted through this route. In addition, our findings suggest it is relatively easier to increase ideational score in this domain compared to the social domain.

## Supplementary Information

**Additional file 1**:**Appendix 1.** Description of psychosocial factors used to create mediator variables.

## Data Availability

The data set used for the preparation of this manuscript is available from the corresponding author upon reasonable request.

## Abbreviations

SBC: Social and behavior change
HC3: Health Communication Capacity Collaborative
IRB: Institutional Review Board
ITN: Insecticide treated bed net
LGA: Local Government Area
NMEP: National Malaria Elimination Programme
NPopc: National Population Commission
PMI: President’s Malaria Initiative
USAID: United State Agency for International Development
WHO: World Health Organization
