# Supplementary material for: The role of ideation on the effect of an SBC intervention on consistent bed net use among caregivers of children under 5 years in Nigeria: a multilevel mediation analysis
Source: BMC Public Health. 2021 Sep 13;21:1660. doi: 10.1186/s12889-021-11709-5 (PMC8436470; doi:10.1186/s12889-021-11709-5)
Supplement: Supplementary file 1 — Additional file 1:Appendix 1. Description of psychosocial factors used to create mediator variables. [file 12889_2021_11709_MOESM1_ESM.docx]

**Appendix 1: Description of how psychosocial factors were measured and manipulated**

| Psychosocial factor | Questions/statements used to assess | Ideational domain | Explanation of responses | Quantification of scores |
| --- | --- | --- | --- | --- |
| Knowledge about the cause of malaria | What causes malaria? | Cognitive | This was a multiple response question and only respondents who list mosquito bite as the only cause of malaria were categorized as having adequate knowledge about the cause of malaria | Respondents who were categorized as having adequate knowledge of the cause of malaria were given a score of 1 and others got a score of 0 |
| Knowledge of a place to purchase bed nets | Do you know of a place in your community where you could purchase a mosquito net? |  | This was a yes or no question and respondents who answered yes were categorized as knowing where to purchase a net | Respondents categorized as knowing where to purchase a bed net were given a score of 1 and others got a score of 0 |
| Knowledge that use of bed net is a way to prevent malaria | What are the things that people can do to stop them from getting malaria? |  | This was a multiple-choice question and people who mentioned “sleep inside a mosquito net” were categorized as knowing that use of bed net is a way to prevent malaria | Respondents categorized as knowing that use of bed net is a way to prevent malaria were given a score of 1 and others got a score of 0 |
| Attitudes towards bed nets | - It only takes a few months for a bed net to get too many holes to stop mosquitoes. - It is difficult to sleep well under a bed net when the weather is warm. - Sleeping under a bed net is a good way to get privacy in a crowded house. - Many people will choose not to sleep under a bed net if they don’t like its color. - It is easier to get a good night’s sleep when you sleep under a bed net. - The insecticide on bed nets can be dangerous to people who sleep under them |  | Six statements were used to assess attitude to bed nets and respondents selected one of five options: strongly agree, somewhat agree, somewhat disagree, strongly disagree or don’t know.  If the expected response to a statement was strongly agree, each respondent who chose strongly agree will get a score of 2, respondents who chose somewhat agree will get a score of 1, respondents who chose somewhat disagree will get a score of -1, respondents who chose strongly disagree will get a score of -2 while those who chose don’t know will get a score of 0.  Conversely, if the expected response was strongly disagree, respondents who chose strongly disagree will get a score of 2, those that chose somewhat agree will get a score of 1, those that chose somewhat agree will get a score of -1, those who chose strongly agree will get a score of -2 and those that chose don’t know will get a score of 0 | The score for each statement used to assess this psychosocial factor ranged between 2 to -2. Therefore, this factor has a score of between 12 and -12 since there are six statements. Given the need to identify respondents with a positive (or higher) attribute for this factor, we split the score at the median and categorized respondents with a score above median as having a positive (higher) attribute and created a new variable with a score of 1 for those with higher attribute and a score of 0 for others. Furthermore, each psychosocial factor can only have a score of 1 or 0 when combining them into the composite variable of ideation. |
| Descriptive norm about bed net use | Generally, in how many households in your community do people sleep under a bed net – All households, most households, at least half of the households, fewer than half households, or hardly any households? |  | This was a multiple-choice question and respondents were expected to select one option. The question was designed to assess what respondents thought people in their community were doing. Respondents who chose all households, most households or at least half the households were categorized as having a favorable norm about net use | Respondents categorized as having a favorable norm were given a score of 1 and others got a score of 0 |
| Perceived severity of malaria, if infected | - You don’t worry about malaria because it can be easily treated. - Every case of malaria can potentially lead to death. - When someone you know gets malaria, you usually expect them to completely recover in a few days. - When your child has a fever, you usually wait a couple of days before going to a health provider. - Only weak children can die from malaria. |  | Five statements were used to assess how serious respondents perceived malaria to be.  Respondents selected one of five options: strongly agree, somewhat agree, somewhat disagree, strongly disagree or don’t know.  If the expected response to a statement was strongly agree, each respondent who chose strongly agree will get a score of 2, respondents who chose somewhat agree will get a score of 1, respondents who chose somewhat disagree will get a score of -1, respondents who chose strongly disagree will get a score of -2 while those who chose don’t know will get a score of 0.  Conversely, if the expected response was strongly disagree, respondents who chose strongly disagree will get a score of 2, those that chose somewhat agree will get a score of 1, those that chose somewhat agree will get a score of -1, those who chose strongly agree will get a score of -2 and those that chose don’t know will get a score of 0 | The score for each statement used to assess this psychosocial factor ranged between 2 to -2. Therefore, this factor has a score of between 10 and -10 since there are five statements. Given the need to identify respondents with a positive (or higher) attribute for this factor, we split the score at the median and categorized respondents with a score above median as having a positive (higher) attribute and created a new variable with a score of 1 for those with higher attribute and a score of 0 for others. Furthermore, each psychosocial factor can only have a score of 1 or 0 when combining them into the composite variable of ideation. |
| Perceived susceptibility to malaria infection | - During the rainy season, you worry almost every day that someone in your family will get malaria. - When your child has a fever, you almost always worry that it might be malaria. - People in this community only get malaria during the rainy season. - People only get malaria when there are lots of mosquitos. - You cannot remember the last time someone you know became dangerously sick with malaria. |  | Five statements were used to assess how vulnerable respondents thought they are to malaria.  Respondents selected one of five options: strongly agree, somewhat agree, somewhat disagree, strongly disagree or don’t know.  If the expected response to a statement was strongly agree, each respondent who chose strongly agree will get a score of 2, respondents who chose somewhat agree will get a score of 1, respondents who chose somewhat disagree will get a score of -1, respondents who chose strongly disagree will get a score of -2 while those who chose don’t know will get a score of 0.  Conversely, if the expected response was strongly disagree, respondents who chose strongly disagree will get a score of 2, those that chose somewhat agree will get a score of 1, those that chose somewhat agree will get a score of -1, those who chose strongly agree will get a score of -2 and those that chose don’t know will get a score of 0 | The score for each statement used to assess this psychosocial factor ranged between 2 to -2. Therefore, this factor has a score of between 10 and -10 since there are five statements. Given the need to identify respondents with a positive (or higher) attribute for this factor, we split the score at the median and categorized respondents with a score above median as having a positive (higher) attribute and created a new variable with a score of 1 for those with higher attribute and a score of 0 for others. Furthermore, each psychosocial factor can only have a score of 1 or 0 when combining them into the composite variable of ideation. |
| Perceived response-efficacy of bed nets (belief that sleeping inside a bed net can prevent malaria infection). | - Your chances of getting malaria are the same whether or not you sleep under a bed net. - Many people who sleep under a bed net still get malaria. - Sleeping under a bed net every night is the best way to avoid getting malaria. - More expensive bed nets are more effective than less expensive or free bed nets. - Bed nets only prevent mosquito bites when used with certain types of beds. - You mainly use a bed net to avoid malaria. |  | Six statements were used to assess respondent’s perception of the effectiveness bed nets in preventing malaria. Respondents selected one of five options: strongly agree, somewhat agree, somewhat disagree, strongly disagree or don’t know.  If the expected response to a statement was strongly agree, each respondent who chose strongly agree will get a score of 2, respondents who chose somewhat agree will get a score of 1, respondents who chose somewhat disagree will get a score of -1, respondents who chose strongly disagree will get a score of -2 while those who chose don’t know will get a score of 0.  Conversely, if the expected response was strongly disagree, respondents who chose strongly disagree will get a score of 2, those that chose somewhat agree will get a score of 1, those that chose somewhat agree will get a score of -1, those who chose strongly agree will get a score of -2 and those that chose don’t know will get a score of 0 | The score for each statement used to assess this psychosocial factor ranged between 2 to -2. Therefore, this factor has a score of between 12 and -12 since there are six statements. Given the need to identify respondents with a positive (or higher) attribute for this factor, we split the score at the median and categorized respondents with a score above median as having a positive (higher) attribute and created a new variable with a score of 1 for those with higher attribute and a score of 0 for others. Furthermore, each psychosocial factor can only have a score of 1 or 0 when combining them into the composite variable of ideation. |
|  |  |  |  |  |
| Perceived self-efficacy to prevent oneself or one’s children from malaria | - You could easily protect yourself from getting malaria - You could easily protect your children from getting malaria | Emotional | Two statements were used to assess this factor and respondent were expected to choose one of five options: Definitely could, probably could, probably could not, definitely could not and don’t know.  If the expected response was definitely could, then respondents who chose definitely could, will get a score of 2, those who chose probably could, will get a score of 1, those who chose probably could not, will get a score of -1, those who chose definitely could not, will get a score of -2 and those who chose don’t know will get a score of 0. | The score for each statement used to assess the psychosocial factor ranged between 2 to -2. Therefore, this factor has a score of between 4 and -4 since there are two statements. Given the need to identify respondents with a positive (or higher) attribute for this factor, we split the score at the median and categorized respondents with a score above median as having a positive (higher) attribute and created a new variable with a score of 1 for those with higher attribute and a score of 0 for others. Furthermore, each psychosocial factor can only have a score of 1 or 0 when combining them into the composite variable of ideation. |
| Perceived self-efficacy to procure and use bed nets | - You could obtain enough bed nets to cover all of the sleeping spaces in your household. - You could sleep under a bed net for the entire night when there are lots of mosquitos.   You could sleep under a bed net for the entire night when there are few mosquitos |  | Three statements were used to assess this factor and respondent were expected to choose one of five options: Definitely could, probably could, probably could not, definitely could not and don’t know.  If the expected response was definitely could, then respondents who chose definitely could will get a score of 2, those who chose probably could will get a score of 1, those who chose probably could not will get a score of -1, those who chose definitely could not will get a score of -2 and those who chose don’t know will get a score of 0. | The score for each statement used to assess the psychosocial factor ranged between 2 to -2. Therefore, this factor has a score of between 6 and -6 since there are three statements. Given the need to identify respondents with a positive (or higher) attribute for this factor, we split the score at the median and categorized respondents with a score above median as having a positive (higher) attribute and created a new variable with a score of 1 for those with higher attribute and a score of 0 for others. Furthermore, each psychosocial factor can only have a score of 1 or 0 when combining them into the composite variable of ideation. |
| Willingness to pay for nets | Would you be willing to pay for a new mosquito net? |  | This was a yes or no question and respondents who chose yes were categorized as being willing to pay for a bed net. | Respondents categorized as willing to pay for a bed net were given a score of 1 and others got a score of 0 |
|  |  |  |  |  |
| Discussion about bed nets with others | In the past year, how often have you discussed malaria with your friends or relations (apart from your spouse) – frequently, sometimes, not very often, never?  What topics have you discussed in the past year? | Social | The first question asked responded if they have discussed malaria. Respondents who indicated that they have discussed malaria irrespective of the frequency of the discussion were asked what topics they discussed.  Respondents who mentioned discussing about bed net in the second question were categorized as having had discussions about bed net. | Respondents categorized as having had discussions about bed net were given a score of 1 while others got a score of 0 |
| Participation in net allocation decisions in the household | In your household, who usually makes decisions about where each bed net will be used – you, your spouse, you and your spouse, or someone else? |  | This was a multichoice question and respondents who indicated that they themselves alone or jointly with their spouse decide bed net allocation, were categorized as participating in net decision allocation. | Respondents categorized as participating in bed net allocation decision were given a score of 1 while others got a score of 0 |
|  |  |  |  |  |
